# A high-starch vs. high-fibre diet: effects on the gut environment of the different intestinal compartments of the horse digestive tract

**DOI:** 10.1186/s12917-022-03289-2

**Published:** 2022-05-19

**Authors:** Federica Raspa, Ingrid Vervuert, Maria Teresa Capucchio, Elena Colombino, Domenico Bergero, Claudio Forte, Martina Greppi, Laura Cavallarin, Marzia Giribaldi, Sara Antoniazzi, Damiano Cavallini, Ermenegildo Valvassori, Emanuela Valle

**Affiliations:** 1 Department of Veterinary Sciences, University of Turin, 10095 Grugliasco, Italy; 2 Faculty of Veterinary Medicine, Leipzig University, 04103 Leipzig, Germany; 3 ISPA-CNR, Institute of Sciences of Food Production, 10095 Grugliasco, Italy; 4 Department of Veterinary Sciences, University of Bologna, 40064 Ozzano dell’Emilia, Italy; 5 Public veterinary service, ASL TO5, 10023 Chieri, Italy

**Keywords:** Horse, Gut environment, Intestinal compartments, Volatile fatty acids, Particle size

## Abstract

**Background:**

Horses are often fed high amounts of starch in their diets despite the well-established benefits of a fibre-based diet to promote gut health and animal welfare. The aim of the present study was to compare the effects of two different diets – one based on high amounts of starch (HS) vs. one base on high amounts of fibre (HF) – on specific parameters of the gut environment across different intestinal compartments of the horse digestive tract. To this end differences in the gastrointestinal environment between HS vs. HF fed horses were assessed in terms of dry matter, organic matter and ash content; the particle size distribution and volatile fatty acid composition were also investigated.

**Results:**

Nineteen Bardigiano horses of 14.3 ± 0.7 months of age and destined to slaughter were divided into two group pens – one fed with high amounts of starch (HS; *n* = 9; 43% hay plus 57% starch-rich pelleted feed); vs. fed with high amounts of fibre (HF; *n* = 10; 70% hay plus 30% fibre-rich pelleted feed). Horses fed HS diet presented a higher dry matter content in the right dorsal colon. Moreover, they showed a higher organic matter and ash content in the sternal flexure, pelvic flexure, right dorsal colon and rectum. In these latter intestinal compartments, horses fed a HS diet also showed a higher proportion of particles retained on an 8 mm sieve and a higher proportion of particles that washed through the finest sieve (< 1 mm). Moreover, the total amounts of volatile fatty acids as well as valeric acid were found to be significantly higher in horses fed the HS vs. HF diet.

**Conclusions:**

A high-starch diet causes significant changes in the horse gut environment. We observed an increase in the dry matter content in the right dorsal colon, as well as reduced particle sizes and an increase in the production of valeric acid in all the gut compartments studied. High-starch diets should be avoided in favour of fibre-based diets with the goal of safeguarding gut health in horses.

## Background

A high-fibre, low starch diet should be promoted to ensure good health and safeguard welfare in horses [1]. However, despite many authors sustaining that starch in the diet should be restricted to no more than 2 g/kg bodyweight (BW)/meal [2, 3], equine feeding regimes – including those of competition or leisure horses as well as horses reared for meat production – are often characterised by high amounts of concentrate feeds, usually based on starch-rich cereal grains mix [4–6]. In fact, cereal grains themselves are rich in starch and their content on dry matter basis depends on the seed considered – i.e. oat contain around 40%, barley around 50 to 55%, and corn around 60% [7].

High starch diets in horses are defined as rations that consist of more than 40% of starch-rich concentrates [8] and that not respected the safe upper limit for starch intake of 2 g/kg BW/meal [2, 3]. Several studies have underlined that feeding horses low forage to concentrate ratios and high amounts of starch constitutes a risk factor for the onset of gastrointestinal disorders such as gastric ulcers and colic [2, 9], metabolic disorders such as acidosis and laminitis [10–12], and may cause changes in the time-budget or behavioural repertoire [13–15]. According to these considerations, it appears that certain diets can predispose an animal to certain diseases and impair gut health. Gut health is a multidimensional concept related to diet that concerns the structure and the functioning of the gastrointestinal barrier, the gut’s microbial profile, and the digestion and absorption of nutrients [16]. Therefore, both diet composition and feeding management are able to influence gut function – i.e. the absorption and digestion of nutrients – by causing alterations to the gut environment in terms of its microbial profile, the volatile fatty acids and the particle size distribution [17, 18].

Accordingly, studying the effects of diet composition on the gastrointestinal environment is important in order to ascertain the role of the diet on the development of diseases [19]. Although diet composition likely affects the health of all the different intestinal compartments, most studies have used faecal samples for their analyses, being easy and non-invasive to collect, meaning that direct evidence on the differential effects of diet on the distinct intestinal compartments remains sparse [20]. Faecal samples could be not representative of the proximal digestive tract; on the contrary in situ assessment of the different gut compartments, could provide information about what happens along the digestive tract [19, 20]. However, at the moment only De Fombelle et al. [21] have characterised the microbial and biochemical profile of different intestinal compartments in horse according to two dietary treatments. Instead, according to our knowledge no studies on particle size distribution are currently available.

On this basis, the present research aims to compare the effects of two distinct diets – one based on high amounts of starch (HS) vs. one based on high amounts of fibre (HF) – on the properties of different anatomic compartments of the horse digestive tract: the small intestine (SI), the apex of the caecum (CAE), the sternal flexure (SF), the pelvic flexure (PF), the right dorsal colon (RDC) and the rectum (RE). The specific variables analysed in each intestinal compartment in horses fed either the HS or HF diet were: dry matter, organic matter and ash content, the particle size distribution, and the volatile fatty acids produced.

Our goal was to obtain data on the changes induced by a high starch diet – a typical dietary mistake made by horse owners – on a variety of parameters linked to equine gut health.

## Results

### Dry matter, organic matter and ash content

The percentage of dry matter (DM) and of organic matter (OM) and ash content (as a percentage of DM) in faecal samples obtained from the different intestinal compartments of horses are summarized in Table 1 according to the dietary treatment received. The statistical analyses for the effect of diet and sex and their interaction are also shown. The DM content in the RDC was significantly affected by dietary treatment (HS vs. HF). Specifically, horses fed the HS diet showed a higher DM content in the RCD compared with horses fed the HF diet (*p* < 0.01). The OM content was significantly higher in SF, PF, RDC and RE in the horses fed the HF diet compared with those fed the HS diet (*p* < 0.01), and a significant diet*sex interaction was found for DM content in the SF (*p =* 0.02). Moreover, the ash content was significantly higher in SF, PF, RDC and RE in the horses fed HS diet compared with horses fed the HF diet (*p* < 0.01). Once again, a significant diet*sex interaction was identified in relation to the SF (*p =* 0.04).

**Table 1:** Comparison between DM, OM and Ash content according to dietary treatments (HS vs. HF)

	HS	HF	*p*-value		
			Diet	Sex	Diet*Sex
DM (%)					
SI	3.94 (0.38)	4.49 (0.40)	0.39	0.13	0.17
CAE	5.33 (0.56)	5.86 (0.35)	0.33	0.75	0.43
SF	11.51 (0.50)	11.19 (0.36)	0.86	0.94	0.27
PF	11.60 (1.09)	9.83 (0.41)	0.23	0.84	0.58
RDC	14.17 (0.28)	11.04 (0.56)	< 0.01*	0.84	0.25
RE	18.18 (0.61)	16.16 (1.30)	0.31	0.09	0.46
OM (%DM)					
SI	n.a.	n.a			
CAE	79.42 (1.29)	82.47 (1.06)	0.11	0.71	0.95
SF	86.25 (84.64–87.59)	88.32 (87.98–88.84)	< 0.01*	0.19	0.02*^a^
PF	82.63 (0.69)	86.66 (0.38)	< 0.01*	0.47	0.29
RDC	82.48 (0.74)	86.83 (0.38)	< 0.01*	0.87	0.82
RE	82.95 (0.82)	88.80 (0.46)	< 0.01*	0.75	0.61
Ash (%DM)					
SI	n.a.	n.a			
CAE	20.57 (1.29)	17.52 (1.06)	0.11	0.71	0.95
SF	13.74 (12.40–15.35)	11.67 (11.15–12.01)	< 0.01*	0.31	0.04*^b^
PF	17.36 (0.69)	13.33 (0.38)	< 0.01*	0.47	0.29
RDC	17.51 (0.74)	13.16 (0.38)	< 0.01*	0.87	0.82
RE	17.04 (0.82)	11.19 (0.46)	< 0.01*	0.75	0.61

Data not normally distributed are expressed as medians (25th–75th percentiles); data normally distributed are expressed as means (SEM)

*HS* high starch, *HF* high fibre, *SI* small intestine, *n.a* not analysed, *CAE* apex of the caecum, *SF* ventral diaphragmatic flexure of the colon, *PF* pelvic flexure, *RDC* right dorsal colon, *RE* rectum. *statistical significance *p* < 0.05

*^a^females HF 88.48 (88.08–88.84)^A^; males HF 88.16 (85.96–89.15)^A^; males HS 87.60 (86.20–87.74)^A^; females HS 85.49 (83.46–86.36)^B^. ^A,B^ *p* < 0.05

*^b^females HS 14.51 (13.65–16.54)^A^; males HS 12.41 (12.27–13.80)^B^; males HF 11.84 (10.85–14.04)^BC^; females HF 11.52 (11.16–11.92)^C^. ^A,B,C^ *p* < 0.05

### Particle size distribution

The results of the particle size analysis obtained by wet sieving faecal samples from each of the nominated intestinal compartments are summarised in Table 2, shown according to the dietary treatment received. Any diet*sex interactions are also shown.

**Table 2:** Comparison of particle size distributions according to dietary treatments (HS vs. HF)

		HS	HF	*p*-value		
				Diet	Sex	Diet*Sex
SI		n.a.	n.a.			
CAE	8 mm	3.41 (2.91–8.58)	2.76 (1.17–4.06)	0.09	0.35	0.70
	4 mm	11.14 (7.39–14.48)	21.15 (10.53–27.93)	0.06	0.89	0.30
	2 mm	7.75 (0.88)	14.31 (1.39)	< 0.01*	0.20	0.94
	1 mm	6.34 (0.65)	7.46 (0.82)	0.17	0.22	0.33
	< 1 mm	69 50 (2.83)	54.33 (3.92)	0.02*	0.87	0.49
	8 mm	4.68 (0.37)	1.86 (0.29)	< 0.01*	0.52	0.52
	4 mm	18.73 (2.39)	23.71 (2.43)	0.24	0.77	0.58
SF	2 mm	11.55 (1.69)	17.92 (2.07)	0.01*	0.54	0.05*^a^
	1 mm	7.48 (0.57)	8.22 (0.64)	0.42	0.82	0.71
	< 1 mm	57.54 (2.92)	48.27 (1.40)	0.01*	0.90	0.23
	8 mm	3.65 (2.31–6.27)	1.34 (1.09–2.47)	0.03*	0.76	0.31
	4 mm	17.85 (1.92)	25.95 (2.64)	0.05*	0.49	0.05*^b^
PF	2 mm	10.58 (1.21)	15.78 (0.78)	< 0.01*	0.39	0.34
	1 mm	7.31 (5.12–11.13)	6.39 (5.83–6.95)	0.75	0.87	0.48
	< 1 mm	59.21 (3.38)	48.97 (1.94)	0.03*	0.52	0.33
	8 mm	4.54 (2.17–5.98)	1.65 (1.01–2.30)	< 0.01*	0.18	0.96
	4 mm	21.70 (1.93)	25.67 (2.14)	0.20	0.26	0.62
RDC	2 mm	1.75 (1.55)	18.14 (1.64)	0.06	0.57	0.85
	1 mm	6.64 (4.94–8.97)	6.78 (6.42–7.46)	0.82	0.75	0.91
	< 1 mm	54.03 (3.37)	47.30 (1.57)	0.12	0.56	0.60
	8 mm	3.75 (0.52)	2.13 (0.27)	0.02*	0.08	0.85
	4 mm	19.28 (1.81)	33.13 (2.76)	< 0.01*	0.67	0.16
RE	2 mm	12.92 (1.21)	17.90 (2.21)	0.07	0.50	0.49
	1 mm	10.29 (1.31)	9.20 (1.76)	0.56	0.41	0.86
	< 1 mm	53.72 (2.29)	37.61 (1.91)	< 0.01*	0.68	0.27

Values are expressed as a percentage (%) of particles, on a dry matter basis, retained by each sieve (8, 4, 2, 1 and < 1 mm)

Data not normally distributed are expressed as medians (25th–75th percentiles); data normally distributed are expressed as means (SEM)

*HS* high starch, *HF* high fibre, *SI* small intestine, *n.a* not analysed according to methods section, *CAE* apex of the caecum, *SF* ventral diaphragmatic flexure of the colon, *PF* pelvic flexure, *RDC* right dorsal colon, *RE* rectum. *statistical significance *p* < 0.05

*^a^males HF 21.2 (13.27–27.67)^A^; females HF 17.72 (14.35–21.82)^A^; females HS 14.84 (12.08–17.46)^AB^; males HS 8.5 (3.48–10.55)^B^. ^A,B^ *p* < 0.05

*^b^females HF 26.04 (18.16–37.86)^A^; males HS 21.72 (19.55–27.58)^AB^; males HF 22.22 (17.81–28.07)^AB^; females HS 14.73 (11.94–15.14)^B^. ^A,B^ *p* < 0.05

In the CAE, dietary treatment significantly affected the particle size distribution. In particular, the proportion of faecal particles retained on the 2 mm sieve was significantly higher in horsing receiving the HF diet compared with those receiving the HS diet (*p* < 0.01). Instead, the fraction of particles that washed through the finest sieve (< 1 mm) was higher in the HS group than in the HF group (*p* = 0.02).

With regard to the SF compartment, differences were found between the two groups related to the proportion of particles retained on the 8 mm sieve, which was higher in horses fed HS diet compared with those on the HF diet (*p* < 0.01). Once again, the proportion of particles retained by the 2 mm sieve was higher in the in HF group compared with the HS group (*p =* 0.01); however, in this case, a significant diet*sex interaction was also present (*p =* 0.05). Finally, a higher fraction of particles washed through the finest sieve (< 1 mm) in samples from the HS group compared with samples from the HF group (*p =* 0.01).

In the PF, similar to the previous compartment along the digestive tract, the SF, the proportion of faecal particles retained on 8 mm sieve was greater in horses receiving the HS diet compared with those on the HF diet (*p =* 0.03). Moreover, in the PF, dietary treatment (HS vs. HF) also had a significant effect on the proportion of particles retained on the 4 mm sieve (*p =* 0.05). In this case, the proportion of faecal particles was higher in horses fed the HF diet than in those receiving the HS diet, and a significant diet*sex interaction (*p* = 0.05) was shown. Once again, the proportion of faecal particles retained on 2 mm sieve in the PF was higher in horses fed the HF diet than those fed the HS diet (*p* < 0.01); whereas the fraction that washed through the finest sieve (< 1 mm) was higher in horses in the HS group (*p =* 0.03).

In the RDC, a significant difference in the fraction of particle sizes retained was observed for the 8 mm sieve, which was higher for faecal samples collected from horses in the HS group (*p* < 0.01).

In the RE, dietary treatment once again significantly affected the proportion of particles retained on the 8 mm sieve (*p =* 0.02). The proportion of particles was higher in horses fed the HS diet than those fed the HF diet. As in the PF, the opposite was true for the 4 mm sieve, for which the proportion of faecal particles retained was greater in horses fed HF diet than in those fed the HS diet (*p* < 0.01. The fraction that washed through the finest sieve (< 1 mm) was once again higher in case of horses fed the HS diet (*p* < 0.01), as occurred in all the other intestinal compartments with the exception of the RDC.

### Volatile fatty acids (VFAs)

Table 3 reports the results of the volatile fatty acid analysis conducted on faecal samples obtained from the distinct intestinal compartments of horses receiving the two dietary treatments, and any diet*sex interactions. The total amounts of VFAs (mg/100 ml) produced in the in the SF, PF, RDC and RE were significantly higher in horses receiving the HS diet compared with those receiving the HF diet (*p* < 0.01); no differences were found in total VFAs between treatment groups for the SI and CAE. Moreover, the percentage (%) of valeric acid on the total VFAs was significantly higher (*p* < 0.01) in horses receiving the HS diet for all the sampled gut compartments – CAE, SF, PF, RDC and RE; conversely, the valeric acid was not detected in the HF group.

**Table 3:** Total VFAs (mg/100 ml) and individual VFAs expressed as a percentage (%) of total VFAs in the different intestinal compartments of the equine digestive tract according to the dietary treatment received (HS vs. HF)

Intestinal compartments	VFAs	HS	HF	*p*-value		
				Diet	Sex	Diet*Sex
SI	Total VFAs	182.82 (130.20–235.04)	176.81 (142.89–238.49)	0.99	0.52	0.90
	Succinic	11.09 (3.00–14.29)	3.86 (0–16.87)	0.29	0.47	0.80
	Lactic	5.23 (3.60–7.83)	5.62 (2.96–26.27)	0.76	0.19	0.46
	Formic	0 (0–1.04)	0 (0–2.59)	0.92	0.05	0.92
	Acetic	55.37 (4.15)	51.32 (5.44)	0.63	0.54	0.65
	Propionic	0 (0–0)	0 (0–0)	0.74	0.26	0.74
	Iso-butyric	24.84 (11.05–32.57)	16.41 (11.63–24.66)	0.55	0.02*^a^	0.50
	Butyric	0 (0–0)	0 (0–0)	1.00	1.00	1.00
	Iso-valeric	0 (0–0)	0 (0–0.80)	0.38	0.38	0.38
	Valeric	0 (0–0)	0 (0–0.88)	0.59	0.64	0.75
CAE	Total VFAs	510.72 (261.05–771.56)	389.17 (290.76–462.55)	0.57	0.95	0.30
	Succinic	0 (0–0)	0 (0–0)	0.84	0.36	0.84
	Lactic	5.90 (2.43–6.84)	3.57 (2.79–6.65)	0.55	0.74	0.66
	Formic	0.50 (0.22–0.91)	0 (0–0.99)	0.10	0.01*^b^	0.97
	Acetic	20.19 (14.00)	30.16 (1.07)	< 0.01*	0.88	0.53
	Propionic	4.19 (3.69–5.31)	9.05 (6.91–10.72)	< 0.01*	0.32	0.79
	Iso-butyric	45.09 (43.05–52.16)	51.84 (47.31–56.48)	< 0.01*	0.11	0.90
	Butyric	2.01 (1.66–2.18)	3.79 (2.85–4.23)	< 0.01*	0.31	0.54
	Iso-valeric	0 (0–0)	0 (0–0)	0.38	0.38	0.38
	Valeric	19.26 (15.00–27.36)	0 (0–0)	< 0.01*	0.64	0.64
SF	Total VFAs	838.11 (622.74–1054.03)	436.14 (381.38–489.42)	< 0.01*	0.87	0.09
	Succinic	0 (0–1.21)	0.50 (0.31–0.95)	0.19	0.35	0.79
	Lactic	5.70 (0.10–6.66)	6.73 (4.88–12.33)	0.06	0.62	0.36
	Formic	0.32 (0.27–1.11)	0.65 (0–1.48)	0.74	0.58	0.47
	Acetic	17.71 (1.51)	33.42 (1.73)	< 0.01*	0.29	0.08
	Propionic	3.80 (2.55–4.44)	8.67 (7.01–9.74)	< 0.01*	0.40	0.63
	Iso-butyric	39.29 (37.77–42.78)	43.90 (36.91–49.68)	0.31	0.34	0.95
	Butyric	2.72 (2.54–3.26)	6.19 (5.35–7.29)	< 0.01*	0.52	0.29
	Iso-valeric	0 (0–0.14)	0 (0–0)	0.51	0.37	0.69
	Valeric	30.74 (26.19–36.94)	0 (0–0)	< 0.01*	0.06	0.06
PF	Total VFAs	897.97 (804.56–1121.135)	303.72 (235.53–342.82)	< 0.01*	0.30	0.50
	Succinic	0 (0–0.68)	0.47 (0–1.42)	0.50	0.52	0.17
	Lactic	1.94 (0.23–3.92)	1.93 (0.90–2.44)	0.92	0.96	0.21
	Formic	0 (0–0.65)	0.23 (0–1.19)	0.51	0.68	0.25
	Acetic	13.93 (1.58)	36.61 (0.75)	< 0.01*	0.27	0.49
	Propionic	2.48 (2.23–3.64)	9.60 (8.08–10.82)	< 0.01*	0.22	0.75
	Iso-butyric	36.44 (32.23–42.31)	47.17 (43.81–48.63)	< 0.01*	0.28	0.04*^c^
	Butyric	2.04 (1.31–2.30)	4.10 (2.54–5.45)	< 0.01*	0.13	0.72
	Iso-valeric	0 (0–0.20)	0 (0–0.47)	0.88	0.94	0.99
	Valeric	43.34 (36.43–43.32)	0 (0–0)	< 0.01*	0.33	0.33
RDC	Total VFAs	835.54 (672.89–1090.51)	284.62 (202.24–340.58)	< 0.01*	0.58	0.41
	Succinic	0.10 (0–0.56)	0 (0–0.60)	0.46	0.10	0.88
	Lactic	0.25 (0–3.29)	3.03 (1.40–3.80)	0.07	0.58	0.60
	Formic	0 (0–0.19)	0 (0–0.21)	0.76	0.63	0.25
	Acetic	15.93 (1.86)	36.31 (2.29)	< 0.01*	0.16	0.72
	Propionic	3.40 (2.73–3.84)	8.92 (6.84–9.46)	< 0.01*	0.40	0.52
	Iso-butyric	39.10 (25.67–41.05)	46.62 (41.49–56.17)	< 0.01*	0.14	< 0.01*^d^
	Butyric	1.78 (1.44–4.16)	3.20 (2.35–4.16)	< 0.01*	0.56	0.98
	Iso-valeric	0.18 (0–0.59)	0 (0–0)	0.06	0.13	0.43
	Valeric	39.25 (36.39–52.21)	0 (0–0)	< 0.01*	0.33	0.33
RE	Total VFAs	605.76 (585.70–916.29)	195.39 (134.52–295.35)	< 0.01*	0.21	0.83
	Succinic	0 (0–1.02)	0 (0–0)	0.08	0.58	0.58
	Lactic	3.04 (0.34–5.89)	3.32 (2.14–4.13)	0.61	0.08	0.28
	Formic	0 (0–0.23)	0 (0–1.06)	0.70	0.08	0.70
	Acetic	13.13 (1.37)	28.82 (1.69)	< 0.01*	0.05	0.71
	Propionic	2.08 (1.93–3.72)	6.61 (5.89–8.80)	< 0.01*	0.49	0.31
	Iso-butyric	37.12 (30.67–52.77)	62.16 (53.42–64.11)	< 0.01*	0.29	0.03*^e^
	Butyric	1.45 (0.79–2.11)	2.00 (0.93–2.60)	0.92	0.17	0.95
	Iso-valeric	0 (0–0)	0 (0–0)	0.26	0.26	0.26
	Valeric	44.59 (19.98–51.79)	0 (0–0)	< 0.01*	0.11	0.11

Data not normally distributed are expressed as medians (25th–75th percentiles); data normally distributed are expressed as means (SEM)

*HS* high starch, *HF* high fibre, *SI* small intestine, *CAE* apex of the caecum, *SF* ventral diaphragmatic flexure of the colon, *PF* pelvic flexure, *RDC* right dorsal colon, *RE* rectum. *statistical significance *p* < 0.05

*^a^males 30.50 (19.19–38.45); females 15.68 (7.97–22.90)

*^b^females 0.71 (0.12–1.33); males 0 (0–0.45)

*^c^males HF 47.99 (46.75–50.55)^A^; females HF 46.44 (40.00–47.66)^A^; females HS 40.78 (34.74–50.79)^AB^; males HS 32.90 (28.32–36.35)^B^. ^A,B^ *p* < 0.05

*^d^males HF 52.79 (47.17–60.11)^A^; females HF 43.19 (38.83–55.10)^A^; females HS 40.92 (35.16–48.41)^A^; males HS 25.67 (18.70–31.67)^B^. ^A,B^ *p* < 0.05

*^e^males HF 63.42 (62.30–64.41)^A^; females HF 57.33 (50.34–60.02)^A^; females HS 37.68 (36.17–66.13)^AB^; males HS 30.67 (23.87–37.92)^B^. ^A,B^ *p* < 0.05

In the CAE, a significantly higher production (%) of acetic acid, propionic acid, iso-butyric acid and butyric acid was detected in the horses fed the HF diet compared with horses fed the HS diet (*p* < 0.01).

In the SF, a significantly higher production (%) of acetic acid, propionic acid and butyric acid was observed in horses fed the HF diet (*p* < 0.01).

In the PF and RDC, acetic acid, propionic acid, butyric acid and iso-butyric acid were produced in significantly higher amounts in horses receiving the HF diet compared with those on the HS diet (*p* < 0.01).

From the most distal intestinal compartment, the RE, higher levels of acetic acid, propionic acid and iso-butyric acid were found in samples from horses fed the HF diet (*p <* 0.01).

A significant diet*sex interaction was shown for iso-butyric acid content in the SF (*p* = 0.04), RDC (*p* < 0.01) and RE (*p* = 0.03).

## Discussion

The present study was carried out in an authorised horse breeding farm for meat production. The farm adopts intensive farming systems characterised by animals kept in pens with no access to pasture [22] and intensive feeding management [6]. The goal of the present study was to evaluate the effects of two different diets – one based on high amounts of starch (HS) vs. one base on high amounts of fibre (HF) – on specific parameters related to gut health within the different intestinal compartments of the horse digestive tract. The DM content and ash content of faecal samples obtained from horses fed high quantities of grains (HS) were both significantly higher compared with samples obtained from horses consuming the HF diet. What it is interesting is that the DM content of the right dorsal colon was higher in the HS group, an effect that was due to diet only, and not sex. This lies in agreement with the findings of Lopes and colleagues [23], who reported that feeding large amounts of grains (4.55 kg per day divided between two rations) reduced water content in the digesta of the right dorsal colon compared with a hay-only diet. Moreover, the same authors found that this level of grain ingestion resulted in marked changes in the right dorsal colon content and in the faeces that were more homogenous, dehydrated, foamy, and dense in comparison with the hay-only diet. The effect on the DM content may be due to different factors. One is related to the fact that feeding meals composed of high amounts of cereal grains causes postprandial dehydration as a consequence of the absorption of water from the colon [24]. Secondly, it is related to the fact that low forage intake causes less water consumption and a lower water content in the colon since eating forage stimulates water consumption and the forage itself holds water within the gastrointestinal tract [23, 24]. According to some authors the increased intake of preserved forages, increases water intake [25]. Even if one limitation of the present study was related to the fact that we were unable to measure water intake we have observed the behaviours of the animals and the horses belonging to HF showed a statistic tendency to drank more than horses belonging to HS (unpublished data). Moreover, the data obtained may support the finding by Lopes and colleagues [23] which stated that high VFA production may lead to greater levels of sodium and water absorption by the colonic mucosa. In fact, we observed significantly higher levels of total VFAs in horses fed the HS diet in all hindgut compartments (i.e. the sternal flexure, pelvic flexure, right dorsal colon and rectum) compared with those fed the HF diet. This finding corroborates those of other studies [21, 26] which show that horses fed diets with a high starch content contain high concentrations of total VFAs across all segments of the intestinal tract.

Acetate, propionate and butyrate are reported to be the primary VFAs produced by bacterial fermentation within the equine gastrointestinal tract. In particular, the percentage of VFAs in caecal or colonic fluid is reported to be approximately 74% acetate, 17% propionate, 6% butyrate and 3–4% other VFAs (isobutyrate, valerate and isovalerate) [24].

A high total VFA content may also increase the risk of digestive disturbances, such as colic, osmotic diarrhea and laminitis [27]. All horses were monitored for all the period until they were slaughtered. They did not show colic or laminitis; but – as already reported in a previous study carried out on horses fed with similar management [6] – also those animals showed faeces not formed.

Moreover, variations in individual VFA produced may also play a role in the pathogenesis of certain symptoms typically associated with a high starch diet. According to the literature, increasing the proportion of cereal grains promotes the production of propionate and lactate at the expense of acetate [17, 28, 29]. Indeed, our results show that the percentage of acetate over total VFAs was lower in the horses fed the HS diet in all the gut compartments studied compared with the values obtained in the HF group. Moreover, our data revealed the percentage of propionate to be lower in horses fed the HS diet compared with those receiving the HF diet. By contrast, the percentage of butyrate produced in the caecum, pelvic flexure and right dorsal colon was higher. Wambacq et al. [28] reported butyric acid to be an end-product of the microbial fermentation of fibre, and proposed that it may promote gut health by increasing the differentiation of colonocytes, and exert an anti-inflammatory effect and modulate oxidative stress.

Changes in the relative proportions of individual VFAs suggest the occurrence of changes in microbial populations according to the type of diet consumed and the intestinal pH [26], both of which should be investigated in further detail. In particular, our study revealed higher amounts of total VFAs in the HS group that were related to a significant increase in valeric acid, whereas no traces of valeric acid were detected in the HF group. Valeric acid represented around 40% of the total VFAs produced in the hindgut of the HS group. The significance and implications of its presence needs to be investigated and is of particular interest, especially considering the fact that this VFA is produced from lactate by lactate-utilizing bacteria following the former’s accumulation in the case of a HS diet, as suggested by Grimm et al. [18]. It is interesting to underline that, according to Nadeau et al. [30], valeric acid has a high lipid solubility and is able to penetrate the mucosa. The same authors also reported that this VFA is important in the pathogenesis of gastric ulcers. Thus, this effect may also be of significance in the hindgut where inflammation processes have often been associated with high starch diets [2, 27].

In the horses fed a grain-rich, and thus starch-rich, diet (HS), we identified a higher ash content than found in horses fed the fibre-rich diet (HF). This was surprising as the ash intake was similar in the two diets (as reported in Table 1, HS = 901.8 g ash as fed and HF = 904.4 g ash as fed). However, the higher ash content in the intestinal tract of horses on the HS diet could be the result of lower amounts of ash absorption in the intestine as a direct consequence of the high amounts of starch fed in the diet [31]. On the contrary, the higher percentage of OM found in the sternal flexure, the pelvic flexure, the right dorsal colon and the rectum in the HF group could be related to the high fibre intake which has been reported to reduce the digestibility of OM [32, 33]. Moreover, a diet*sex interaction was found in relation to the percentage of OM and ash content in the sternal flexure. The effect of sex was only seen in one of the intestinal compartments analysed, and more research is required to understand the basis of this observation.

To the best of our knowledge, the present study is the first to investigate differences in particle size distribution across the different compartments of the equine intestinal tract according to the diet consumed (HS vs. HF). For faecal samples obtained from the caecum of horses in the HS group, the fraction of particles that washed through the finest sieve (< 1 mm) constituted 69.50% of the digesta. This finding is particularly interesting if we consider that the CAE is one of the most common sites – together with the ileum and the large colon pelvic flexure (PF) – of gastrointestinal tract obstruction or faecal impaction [34–36]. Moreover, in the sternal flexure, the pelvic flexure, the right dorsal colon and the rectum, our results showed that the proportions of faecal particles retained by a 8 mm sieve and washed through the finest sieve (< 1 mm) were higher in horses fed the HS diet compared with those fed the HF diet. The finest particles made up around 50% of the total in the HS group, and this result could explain a finding by Lopes et al. [23], who described the digesta and faeces from horses fed a high starch diet to be more homogenous and dense compared with those from horses fed a hay only diet. In the literature, the majority of studies evaluating faecal particle size relate their findings to the dental status of the horse [37–39]. However, none of the horses involved in our study were affected by dental issues, being young healthy animals, so we must conclude that the differences found were related to the differences in the diets (HS vs. HF). Thus, our results suggest that the particle size is not only influenced by chewing and the condition of the dental board but also by the amounts of starch supplied in the diet. In fact, it is well known that high amounts of undigested starch are responsible for alterations or shifts in microbiome composition, which lead to a reduction in the activity of fibrolytic microorganisms [18, 19, 40] and, as a consequence, a reduction in the fermentation capacity of the fibre [40, 41]. This aspect seems particularly important if we consider that the adequate digestion of fibre is believed to be crucial for reducing particle retention in the intestine, the occurrence of which increases the risk of large colon impaction [36].

## Conclusion

According to our results, a high starch diet has a profound effect on the horse’s gut environment in terms of DM, VFA production and particle sizes. The results of this study confirm the notion that diet composition, and thus feeding management practices, are able to influence the gut environment and its functioning. Importantly, the observed alterations were primarily localized in the sites that colic surgeries frequency reveal to be associated with digestive problems.

Considering what stated above, a fibre-based diets should be promoted in horses to avoid causing alterations in DM, VFAs and particle size composition in order to safeguard gut health in horses.

## Methods

The present study is part of a larger project aimed at improving the welfare of horses reared for meat production through changes to their feeding management. The project compares the effects of a standard feeding regime, adopted by intensive farming systems rearing horses for meat purposes, based on a high starch diet (HS) with a novel feeding regime strategy based on a high fibre diet (HF). The project was organised to study the effects of the two diets on: welfare [6], behavioural activities [14], meat production [42] and gut health.

To avoid the repetition of data that has already been published, a brief summary of the experimental trial is reported below; further details can be found in Raspa et al. 2021 [42]. The trial was approved by the Ethical Committee of the Department of Veterinary Sciences of the University of Turin (Italy, Prot. n. 2202/2019).

### Animals, management and diet

Nineteen Bardigiano horses (12 females, 7 males) of bodyweight (BW) 346 ± 5.2 kg, aged 14.3 ± 0.7 months (mean ± standard deviation, SD) and destined for slaughter, were used in this study. The horses received the same first-cut meadow hay but different complementary feeds. One group of 9 horses was fed a starch-rich complementary feed (HS; 43% hay plus 57% cereal grain-based pelleted feed) characterised by 11.45 g of starch/kg BW/day. A second group of 10 horses was fed a fibre-rich complementary feed (HF; 70% hay plus 30% pelleted fibrous feed) characterised by 1.94 g of starch kg/BW/day. Horses were individually fed the complementary feeds, which were supplied twice a day, and hay was provided estimating its consumption at approx. 6 kg/animal/day for the HS group and 8 kg/animal/day for the HF group. The complementary feeds were gradually increased to reach the final amount during the last 72 days of the fattening period; more details about the feeding trial are reported in Raspa et al. [42]. The proximate analysis of the hay, the different complementary feeds used and the daily nutritional composition of the diets (HS and HF) are reported in Table 4.

**Table 4:** Chemical composition of hay, feed concentrates and overall daily diets (hay plus feed for HS vs. HF)

Items	Hay^a^	Starch-rich feed^a^	Fibre-rich feed^a^	HS diet^b^	HF diet^b^
Dry matter	89.8	89.9	90.6	12.6	10.3
Ash	62.3	83.0	107.8	901.8	904.4
Crude protein	66.2	142.1	197.7	1557.2	1159.6
Ether extract	10.3	36.9	50.6	285.4	192.7
Crude fibre	30.0	44.4	115.3	2258.4	2873.9
Starch	n.a.	495	191.1	3960	669
Net Energy	3.6	9.3	7.2	95.8	53.6

^a^Items are given as g/kg as fed, except dry matter, given as g/kg, and Net Energy, given as MJ/kg as fed

^b^Items are given as g as fed, except dry matter, given as kg as fed, and Net Energy, given as MJ as fed

*n.a* not analysed

### Sample collection

At the end of the fattening period, horses were slaughtered according to the European Union regulations (EU Regulation 2009/853 and EU Regulation 627/2019).

**Fig. 1:**
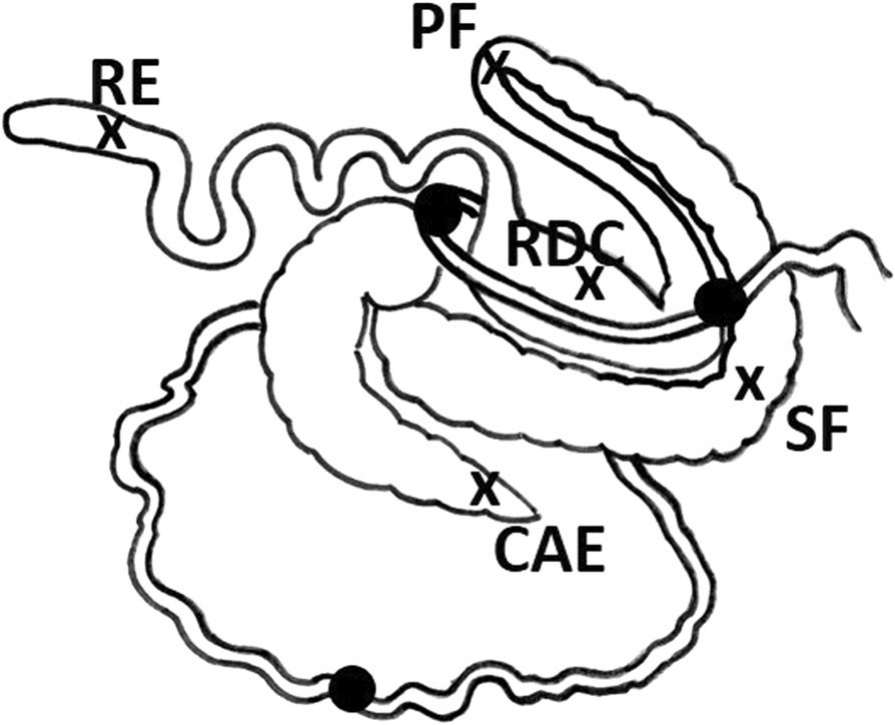
Illustration of the sampling sites. Filled circles indicate the collection sites from the small intestine, which were then pooled to produce a single sample for the duodenum, jejunum and ileum. Crosses indicate the collection sites for the following intestinal compartments: CAE = apex of the caecum, SF = sternal flexure, PF = pelvic flexure; RDC = right dorsal colon; RE = rectum

Immediately after slaughtering, samples of the gut content were taken from different intestinal compartments. The intestinal compartments sampled were the following (see Fig. 1):

- the small intestine (SI) – a pooled chyme sample from the duodenum, jejunum and ileum was collected as a consequence of the fact that there was too little material available from the individual intestinal compartments;
- the apex of the caecum (CAE);
- the sternal flexure (SF);
- the pelvic flexure (PF);
- the right dorsal colon (RDC);
- the rectum (RE).

Each selected intestinal compartment was identified and clamped with ligatures before being opened for sampling. Samples were collected and stored according to the requirements of the various laboratory analyses, as described in the sections below.

### Dry matter, organic matter and ash content analyses

The samples from each intestinal compartment (300 ± 50 g fresh matter) were collected into pre-labelled plastic boxes, which were then sealed and frozen and stored at − 20 °C until analysis. Samples were thawed and dried in a forced-draft oven at 100 °C for 1 h, then at 60 °C until constant weight was obtained. Dried samples were ground to pass a 1 mm sieve, and the dry matter, organic matter and ash content ascertained according to the methods stipulated by VDLUFA [43]. The percentage of organic matter (OM), with respect to DM, in each sample was calculated according to the formula: OM = 100-Ash-Moisture.

### Analysis of particle size

Samples (50 g) from the each of the specified intestinal compartments – SI excluded since the compartment did not contain enough material – were collected into Falcon collection tubes (Falcon Conical Centrifuge Tubes, Tewsbury, MA), sealed and frozen and stored at − 20 °C until analysis.

Particle size was determined by wet sieving according to the method described by Vondran et al. [9]. Briefly, samples were thawed and soaked in beakers containing 1 L water overnight prior to sieving. Samples were passed through sieves of the following mesh sizes for 5 minutes: 8, 4, 2 and 1 mm. The material remaining on each sieve was dried at 60 °C for 12 hours, then cooled before weighing. The dry amount on each sieve was expressed as a percentage of the dry weight of the total sample. The latter was calculated from the weight of the sampled faeces measured before and after drying. The fraction that washed through the finest sieve (< 1 mm) was calculated from the total sample weight minus the sum of the four sieve fractions.

### Analysis of volatile fatty acids (VFAs)

Following slaughtering, faeces samples for VFA quantification were collected into Falcon collection tubes (Falcon Conical Centrifuge Tube, Tewsbury, MA) from the small intestine (pooled sample) (duodenum, jejunum and ileum) and the hindgut compartments. Samples were immediately frozen and stored at − 20 °C until analysis. VFA quantification was carried out according to the methods described by Guantario et al. [44]. Briefly, samples from each small intestine (15 g) and hind gut (30 g) compartment were suspended in 50 and 100 ml of 0.1 N H_2_SO_4_ solution, respectively, homogenized in a stomacher (Lab-Blender 400, Seward, Worthing, UK) for 5 minutes and centrifuged twice at 15,000 X g for 10 min at 4 °C. The resulting extracts were filtered through a paper filter and then through a 0.22 μm pore syringe filter.

High performance liquid chromatography (HPLC) was conducted using a Dionex Ultimate 3000 (Thermo Fisher) with autosampler equipped with a 300 × 7.8 mm Aminex HPX-87H (Bio-Rad) and a guard-column. Injected samples (30 μL) were isocratically separated in 0.005 N H_2_SO_4_ at a flow rate of 0.6 mL/min at 41 °C. VFAs were detected by UV light at 210 nm and identified using an external standard curve (4.95–148.5 mg/100 ml succinic acid; 9–270 mg/100 ml lactic acid; 10.5–314.4 mg/100 ml acetic acid; 9.85–285.5 mg/100 ml propionic acid; 9.4–282.1 mg/100 ml butyric acid; 9.5–285.1 mg/100 ml isobutyric acid; 9.1–273.4 mg/100 ml iso-valeric acid; 9.1–273.2 mg/100 ml valeric acid) created using standards dissolved in 0.1 N H_2_SO_4_. Total VFAs were expressed as mg/100 ml. Individual VFAs were expressed as a percentage (%) of the total VFAs.

### Statistical analysis

Data were statistically analysed using JMPpro v16 software (SAS Institute Inc., Cary, NC). Each parameter was tested for normality using the Shapiro-Wilk test and normalized, when necessary, by box-cox transformation [45]. A linear mixed-effects model was constructed in which dietary treatment, sex and their interaction were set as the model’s fixed effects. Each horse within each sex and diet group was considered an experimental unit and used as the random variable for all analyses. Least squares means were separated using T-Student’s adjusted *p*-values when at least one tendency F-test (*p* ≤ 0.10) was detected in the fixed effect interaction term [46, 47].

## Data Availability

The datasets generated and/or analysed during the current study are not publicly available due to the fact that they are part of a larger ongoing research project with additional data that will be published in the future but are available from the corresponding author on reasonable request.

## Abbreviations

HS: High starch
HF: High fibre
SI: Small intestine
CAE: Apex of the caecum
SF: Sternal flexure
PF: Pelvic flexure
RDC: Right dorsal colon
RE: Rectum
DM: Dry matter
OM: Organic matter
VFAs: Volatile fatty acids
